# IFNL3 genotype is associated with pulmonary fibrosis in patients with systemic sclerosis

**DOI:** 10.1038/s41598-019-50709-9

**Published:** 2019-10-16

**Authors:** Mayada Metwally, Khaled Thabet, Ali Bayoumi, Mandana Nikpour, Wendy Stevens, Joanne Sahhar, Jane Zochling, Janet Roddy, Kathleen Tymms, Gemma Strickland, Susan Lester, Maureen Rischmueller, Gene-Siew Ngian, Jennifer Walker, Pravin Hissaria, Olfat Shaker, Christopher Liddle, Nicholas Manolios, Lorenzo Beretta, Susanna Proudman, Jacob George, Mohammed Eslam

**Affiliations:** 1 Storr Liver Centre, Westmead Institute for Medical Research, Westmead Hospital and University of Sydney, Sydney, NSW Australia; 2 Department of Biochemistry, Faculty of Pharmacy, Minia University, Minia, Egypt; 3 Department of Rheumatology St Vincent’s Hospital (Melbourne), 41 Victoria Parade, Fitzroy, 3065 Victoria Australia; 4 Department of Medicine, The University of Melbourne at St Vincent’s Hospital (Melbourne), 41 Victoria Parade, Fitzroy, 3065 Victoria Australia; 5 Department of Rheumatology, Monash Health, 246 Clayton Road, Clayton, Victoria 3168 Australia; 6 Department of Rheumatology, The Menzies Research Institute of Tasmania, Private Bag 23, Tasmania, 7001 Australia; 7 Department of Rheumatology, Fiona Stanley Hospital (Perth), 11 Robin Warren Drive, Murdoch, 6150 Western Australia Australia; 8 Canberra Rheumatology, Canberra, ACT Australia; 9 Barwon Rheumatology Services, Victoria, Australia; 10 Rheumatology Unit, The Queen Elizabeth Hospital (Adelaide), 28 Woodville Rd, Woodville, 5011 SA Australia; 11 Rheumatology Unit, Flinders Medical Centre (Adelaide), Flinders Drive, Bedford Park, 5042 South Australia Australia; 12 Departments of Clinical Immunology and Immunopathology, Royal Adelaide Hospital, South Australia Adelaide, Australia; 13 Medical Biochemistry and Molecular Biology Department, Faculty of Medicine, Cairo University, Cairo, Egypt; 14 Rheumatology Department, The University of Sydney, Westmead Hospital, Westmead, NSW 2145 Australia; 15 Referral Center for Systemic Autoimmune Diseases, University of Milan and Fondazione IRCCS Ospedale Maggiore Policlinico, Mangiagalli e Regina Elena, via Pace 9, I-20122 Milan, Italy; 16 Rheumatology Unit, Royal Adelaide Hospital (Adelaide), Port Rd, Adelaide, SA 5000 Australia; 17 Discipline of Medicine, University of Adelaide (Adelaide), North Terrace, Adelaide, SA 5000 Australia; 18 Department of Medicine, Monash University (Melbourne), Wellington Rd, Clayton, 3168 Victoria Australia; 19 Immunology, Allergy and Arthritis Department, Flinders University (Adelaide), Sturt Road, Bedford Park, 5042 South Australia Australia

**Keywords:** Genetics research, Translational research

## Abstract

Fibrosis across different organs and tissues is likely to share common pathophysiological mechanisms and pathways. Recently, a polymorphism (rs12979860) near the interferon lambda gene (*IFNL3*) was shown to be associated with fibrosis in liver across multiple disease etiologies. We determined whether this variant is a risk factor for pulmonary fibrosis (PF) and worsening cutaneous fibrosis in systemic sclerosis (SSc). Caucasian patients with SSc (n = 733) were genotyped to test for association with the presence of PF and worsening of skin fibrosis. Serum IFN-λ3 levels from 200 SSc cases were evaluated. An association of the *IFNL3* polymorphism with PF was demonstrated (OR: 1.66 (95% CI: 1.142–2.416, p = 0.008). The *IFNL3* variant was not a risk factor for worsening of skin fibrosis. Functionally, IFN-λ3 serum levels were higher among subjects with PF compared to those unaffected (P < 0.0001). In conclusion, IFNL3 serum levels and the genetic variant known to be associated with liver fibrosis are similarly linked to PF, but not to worsening of skin fibrosis in SSc. These data highlight both common fibrosis pathways operating between organs, as well as differential effects within the same disease.

## Introduction

Despite contributing up to 45% of deaths in the developed world, fibrotic diseases have largely been overlooked from the perspective of a shared phenotype^1^. In this regard, organs such as lung, liver, skin, and kidney, while possessing unique tissue-specific fibrosis features, are likely to share common core and regulatory pathways. Hence, a multi-organ approach was recently suggested to identify druggable targets for generic anti-fibrotic therapies^1^.

Systemic sclerosis (scleroderma, SSc) is a complex autoimmune disease characterized by fibrosis in skin and internal organs, immune dysregulation, and vasculopathy that is associated with a high morbidity and mortality. A hallmark of SSc is clinical and inter-individual heterogeneity^2^. Though the pathogenesis of SSc remains poorly understood, there is accumulating evidence suggesting that at least in part, genetic factors are involved^2^.

Pulmonary fibrosis (PF) (interstitial lung disease; ILD) is a leading cause of morbidity and mortality in patients with SSc^3^. As new treatments become available or are developed for SSc-associated PF and other forms of PF, early identification of high-risk patients is paramount for early intervention and through this, improved clinical outcomes^3^. Unfortunately, reliable predictors of who will develop PF among those with scleroderma are not available. Genetic variants that might contribute to more severe fibrosis and/or progression could fill this need, as they do not fluctuate over the course of disease and are not time-dependent. Hence these variants are attractive candidates for the development of biomarkers predictive of progression and for risk stratification. The latter is also pivotal for personalization of therapy, and for improving clinical trial design for new therapies^2^.

We and others recently reported that single nucleotide polymorphisms (SNPs) in the interferon lambda (*IFNL*, a type III interferon) 3 region originally discovered through genome-wide association studies (GWAS) as a predictor of hepatitis C virus (HCV) clearance, are a strong predictor of the risk for fibrosis risk across multiple liver diseases^4–6^. *IFNL* receptors have restricted expression to epithelial tissues including lung and liver and thus respond to type III IFNs^7^. Thus, it is plausible that genetic variants in the *IFNL3* locus could also affect PF risk. A key difference between idiopathic pulmonary fibrosis (IPF) and SSc-associated PF is the role of inflammation. Inflammation is thought to play a role in SSc-associated PF^2^ but this remains less clear for IPF. In this study, we evaluated whether there is a link between *IFNL3* variants and the risk of skin and pulmonary fibrosis in a large cohort of Caucasian patients with SSc. Functionally, IFNλ-3 levels according to lung fibrosis were also evaluated in human and mice.

## Results

### Patient characteristics

Supplementary Table 1 summarises the main features of the cohort. A total of 733 patients were eligible, of whom 24.5% had PF. Genotyping was successful for all samples except three. The genotype distribution of *IFNL3* rs12979860 conformed to Hardy-Weinberg equilibrium and the minor allele frequency (MAF) was 0.316, similar to that observed in a healthy Caucasian population from the 1000 genome project (http://browser.1000genomes.org). Hence, suggestive that *IFNL3* rs12979860 is not associated with SSc susceptibility.

### *IFNL3* rs12979860 and pulmonary fibrosis

The major *IFNL3* rs12979860 CC genotype previously associated with liver fibrosis was present at a significantly higher frequency in SSc patients with pulmonary fibrosis compared to those without (29% vs 21%, OR: 1.51 (95% CI: 1.077–2.119, p = 0.01). In multiple logistic regression analysis adjusting for age, gender, baseline disease duration and baseline modified Rodnan skin thickness score (mRSS), *IFNL3* rs12979860 CC genotype remained independently associated with the risk of PF (OR: 1.66 (95% CI: 1.142–2.416, p = 0.008). No difference in disease duration was observed between subjects with and without PF or according to *IFNL3* genotype (9.46 (3.2–17.36) vs 8.63 (2.46–18.45), p = 0.6 and (6.7 (1.93–17.6) vs 7.34 (1.72–14.09), p = 0.9) in subjects with CC and CT/TT genotype, respectively.

### *IFNL3* rs12979860 and worsening of skin fibrosis

Next, we evaluated the association of *IFNL3* rs12979860 with worsening of skin fibrosis within ~1 year of study enrolment (follow-up time of 1.07 (0.99–1.36) years). In the overall cohort (only 632 patients had follow-up mRSS recorded). There was no significant association between rs12979860 genotype and worsening of skin fibrosis within 1 year (OR: 0.938, 95% CI: 0.543–1.619, p = 0.8). A similar result was obtained when a Cox proportional-hazards regression model was applied after adjustment for age, sex and baseline disease duration (adjusted HR for time to mRSS > 5: 0.934, 95% CI: 0.458–1.591, p = 0.8). In three further analyses, we considered only subjects with mRSS ≥ 7 (n = 316) at first visit. This cut-off was chosen based on previous reports^8^, that it represents the lowest value required to be considered as diffuse cutaneous systemic sclerosis (dcSSc). In this analysis, rs12979860 had no impact on the risk of worsening of skin fibrosis (OR: 1.13, 95% CI: 0.505–2.531, p = 0.7). In the second analysis conducted in subjects with diffuse SSc (n = 155), rs12979860 again demonstrated no association with the risk of worsening of skin fibrosis. Finally, in a third analysis restricted to subjects with early disease (that is, baseline disease duration shorter than 5 years) (n = 179), *IFNL3* rs12979860 was again not associated with the extent of skin involvement (diffuse vs. limited) or baseline mRSS. No association was observed between IFNL3 genotype and autoantibody status, namely anti-topoisomerase antibody (anti-Scl-70), anti-centromere antibodies or anti-RNA polymerase III (data not shown).

### Serum IFN-λ3 levels

**Figure 1:**
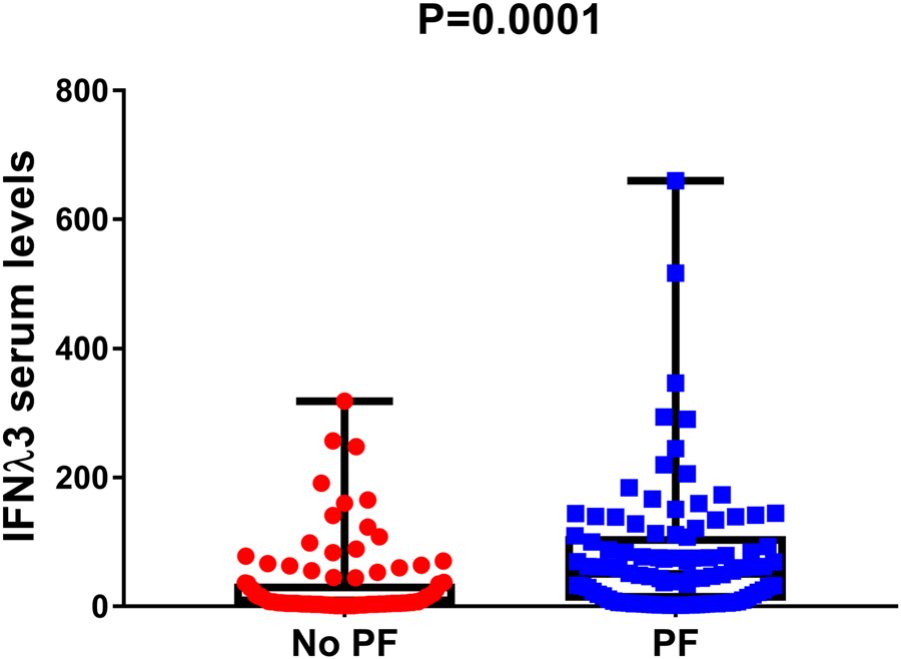
Quantification of IFNλ3 using a highly sensitive chemiluminescent enzyme immunoassay in human serum samples. The levels of IFNλ3 were measured in 200 patients with SSc by a chemiluminescent enzyme immunoassay relative to the presence or absence of pulmonary fibrosis (PF). The x axis shows pulmonary fibrosis status dichotomized as absent (n = 100) or present (n = 100). The y axis shows serum IFNλ3 levels as pg/mL. The number of independent samples tested in each group is shown in parentheses. Each group is shown as a box plot and the median values are shown as thick dark horizontal lines. The box covers the twenty-fifth to seventy-fifth percentiles. We tested the difference in median values among genotypes using the two-tailed Mann–Whitney test and plotted the box plots using Graph pad prism 7.

We recently showed that IFN-λ3 but not IFN-λ4 mediates the *IFNL3/IFNL4* haplotype dependent association with liver inflammation and fibrosis^9^. Hence, to further explore the functional relevance of rs12979860, we evaluated serum IFNλ-3 levels in 200 cases with SSc. As expected and consistent with our hypothesis, IFN-λ3 protein levels were 10 times as high among subjects with PF as among those unaffected by PF (P < 0.0001) (Fig. 1). Importantly, this difference remained significant even after stratification according to rs12979860 genotype (Supplementary Figure 1). Subjects who were homozygous for the major allele have higher serum IFN-λ3 levels compared to those carrying at least one copy of the minor allele in the entire ELISA sub-cohort (median 23.48 (4.28–77.5) vs. 18.35 (4.44–68.3), p = 0.2) and after stratification according to patients with and without pulmonary fibrosis), but this was not significant (Supplementary Figure 2). Notably, 62 (31%) of these patients received one or more immunosuppressive agents. Thus, we examined whether immunosuppressive agents have any effect on IFN-λ3 levels. In this analysis, there was no association between use of immunosuppressive drugs and serum IFN-λ3 levels (median 23.84 (4.29–70.26) vs. 14.2 (4.32–78), p = ns) or between type of immunosuppressive agent when assessed separately. The association between PF and IFN-λ3 levels remained significant after adjusting for the use of immunosuppressive agents (OR = 1.01, 95% CI: 1.004–1.015, p = 0.0001).

### Ifnl3 expression in bleomycin-induced pulmonary fibrosis

**Figure 2:**
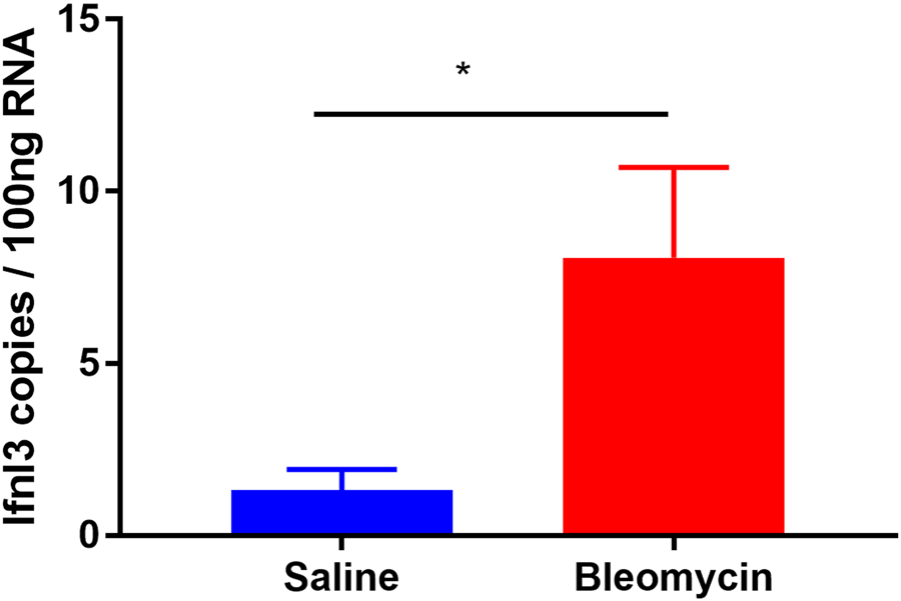
Absolute quantification of Ifnl3 mRNA by ddPCR in murine lung. Basal Ifnl3 expression was measured as number of copies/100 ng of total RNA in lungs of bleomycin- and saline-treated mice by ddPCR analysis. N = 5 mice per each group. Bars represent mean ± SEM. *P < 0.05.

To further investigate the role of IFNL3 in lung fibrosis, we examined Ifnl3 mRNA expression in mice with lung fibrosis induced by bleomycin using ddPCR. Consistent with the human data, Ifnl3 mRNA expression in bleomycin treated mice was significantly higher than that in the control group (P < 0.05) (Fig. 2).

## Discussion

In this study, we demonstrate an association of genetic variants around the *IFNL3* locus with an increased risk of SSc-associated PF, but not with the extent or worsening of skin fibrosis. Furthermore, IFN-λ3 serum levels were higher in subjects with PF compared to unaffected patients, including when stratified by *IFNL3* genotype and in the lungs of mice with bleomycin-induced pulmonary fibrosis. Collectively, our findings in concert with earlier reports on the risk of fibrosis across multiple liver diseases^4,5^, suggest that IFNL3 is a core regulatory fibrotic pathway.

The functional mechanisms for the IFNL3 association with fibrosis generally and PF in particular, remain elusive. There is mounting evidence supporting a role for IFNL in the control of inflammation in response to viral and bacterial infections of epithelial tissues, including of the liver, the gastrointestinal tract and lung^7^. Thus, it is conceivable that IFNL, through its contribution to tissue inflammation that is well-known to be present in the lungs of patients with SSc and PF^2^, modulates the progression of pulmonary disease. Support for a role for IFNL in PF can be extrapolated from other published reports. For example, a recent report showed higher serum IFNL1 levels in patients with SSc compared to healthy controls (11). Further, a variant in the interferon regulator factor-5 (*IRF5*) gene, a transcription factor relevant to the expression of type Ι and Ш interferons was discovered by GWAS as a susceptibility variant for SSc^10^. This was shown later to be associated with better preservation of lung function, milder interstitial lung involvement and longer survival^11^. The protective allele of this variant (*rs4728142*) results in reduced IRF5 transcript levels and presumably IFNL transcription. Consistently, attempts to treat patients with SSc with another IFN (IFN-α) resulted in a substantial worsening of lung disease^12^.

From a clinical perspective, the present findings if further independently replicated, would suggest that *IFNL3* variant testing (either alone or as part of a polygenic risk score) and subsequent risk stratification, could be part of the counselling of patients with SSc who develop PF. From a translational standpoint, there are ongoing efforts to test the safety and efficacy of IFN-α blockade, such as anti-IFN-α receptor monoclonal antibody therapy for the management of SSc^13^. However, due to the restricted expression of IFNL receptors compared to the ubiquitous expression of IFN-α^14^, IFNL blockade could be a more attractive therapeutic target, especially for SSc-associated lung disease.

In this study, *IFNL3* genotype had no impact on the prediction of progressive skin fibrosis in the overall cohort or in the subset with diffuse SSc. As the ASCS cohort is not an inception cohort, the majority of subjects had long disease duration. Since it is well-known that skin thickening progresses more rapidly in early than late disease^2^, we are not able to totally exclude the possibility that inclusion of subjects with longer disease duration might have limited our ability to observe an effect of *IFNL3* genotype on worsening of skin fibrosis. However, our sensitivity analysis in subjects with early disease duration did not show a trend for the association, which argues against this possibility.

The current study has strengths and limitations. The data were derived from a large well-characterised multicenter prospective cohort showing the typical features of SSc with extensive clinical data and disease characteristics. Furthermore, the ASCS-derived dataset enabled analysis of worsening skin fibrosis over a median 12 months follow-up. The latter is widely considered ideal to detect meaningful changes and is used in clinical trials^8^. Limitations of this study include the lack of a replication cohort for the positive association observed with PF, the number of patients with full auto-antibody profiles, and the fact that it was not an inception cohort. Nevertheless, the associations we observed are similar to those previously demonstrated for liver in large independently-replicated cohorts (>4000 and 1300 patients)^4,5^. Further studies will be required to address the impact of IFN-λ3, as well as *IFNL3* genetic variation on ILD severity and progression in cohorts of incident patients followed prospectively with multiple HRCTs, and to clarify the functional mechanisms of this genetic association.

In conclusion, we demonstrate that the *IFNL3* genetic variant and serum levels of IFNλ-3 are significantly associated with the risk of PF in a Caucasian population with SSc. The *IFNL3* variant was not a risk factor for the worsening of skin fibrosis. Consistently, Ifnl3 mRNA expression was higher in lungs from mice with bleomycin-induced pulmonary fibrosis. The functional mechanisms for the pro-fibrotic effects of IFNL3 will require further investigation and if confirmed, can be exploited as a potentially more effective therapeutic target.

## Methods

### Patient cohort

The study comprised 733 consecutive Caucasian patients with SSc from the Australian Scleroderma Cohort Study (ASCS), a multi-center prospective study of risk factors for clinically important outcomes in SSc. All patients fulfilled either American College of Rheumatology or Leroy and Medsger criteria for SSc^14^. The study protocol was conformed to the ethical guidelines of the 1975 Declaration of Helsinki. Each of the participating centres obtained approval for the study from their respective ethics committees and study was performed according to the recommendations of the centers involved. The ASCS compromises 13 Australian centres and has been approved by the human research ethics committee of each of the participating hospitals (St. Vincent’s Hospital, Melbourne Royal Adelaide Hospital, Monash Medical Centre, Royal Perth Hospital, The Queen Elizabeth Hospital, Sunshine Coast Rheumatology, Prince Charles Hospital, John Hunter Hospital, Royal North Shore Hospital, Royal Prince Alfred Hospital, St George Hospital, Canberra Rheumatology and the University of Tasmania). Written informed consent for genetic testing was obtained from all participants. Details of the methods for pulmonary disease assessment, skin fibrosis progression assessment, genotyping, IFN-λ3 serum levels measurement, and the mouse model is provided in Supplementary Methods.

### Statistical analysis

The Student’s *t*-test, Mann-Whitney test, Wilcoxon’s test and Fisher’s exact tests were used, as appropriate. Multiple logistic and Cox proportional-hazards regression models were fitted adjusting for common risk factors as covariates. The *IFNL3* rs12979860 genotype was dichotomized as CC versus CT/TT, as previously reported^4,15^.

## Supplementary information

Supplementary table
