# Supplementary material for: IFNL3 genotype is associated with pulmonary fibrosis in patients with systemic sclerosis
Source: Sci Rep. 2019 Oct 16;9:14834. doi: 10.1038/s41598-019-50709-9 (PMC6795812; doi:10.1038/s41598-019-50709-9)
Supplement: Supplementary file 1 — Supplementary table [file 41598_2019_50709_MOESM1_ESM.docx]

**IFNL3 genotype is associated with pulmonary fibrosis in patients with systemic sclerosis**

Mayada Metwally, Khaled Thabet, Ali Bayoumi, Mandy Nikpour, Wendy Stevens, Joanne Sahhar, Jane Zochling, Janet Roddy, Kathleen Tymms, Gemma Strickland, Susan Lester, Maureen Rischmueller, Gene-Siew Ngian, Jennifer Walker, Pravin Hissaria, Olfat Shaker, Christopher Liddle, Nicholas Manolios, Lorenzo Beretta, Susanna Proudman, Jacob George, Mohammed Eslam

**Supplementary methods:**

**Pulmonary disease assessment**

Pulmonary fibrosis was categorised based upon the results of standard investigations. This was assessed by clinical examination and pulmonary function testing and high resolution computed tomography (HRCT) if ILD was suspected. Thus, lung fibrosis was confirmed by high resolution computed tomography (HRCT) with fibrotic change as previously reported ([1](#_ENREF_1)). Autoantibody status was assessed using commercially available assays.

**Skin fibrosis progression assessment**

Diffuse SSc was characterised by the presence of skin thickening, proximal as well as distal to the elbows and knees, with or without involvement of the face ([2](#_ENREF_2)). Disease duration was estimated as time since first non-Raynaud disease manifestation to baseline visit. Worsening of skin fibrosis was defined as an increase in the modified Rodnan skin thickness score (mRSS) by >5 points from baseline to the 2^nd^ visit within 1 year, which is considered clinically meaningful ([3](#_ENREF_3)). The follow-up period of 12 months was chosen since it is considered a relevant time frame to detect significant changes in mRSS and is therefore used in clinical trials. Data on follow up mRSS was available for 632 subjects.

**Genotyping**

Genotyping for *IFNL3* rs12979860 was contracted to the Australian Genome Research Facility (AGRF; QLD, Australia). Samples were genotyped using the Sequenom MassARRAY system and iPLEX Gold chemistry, or using the TaqMan SNP genotyping allelic discrimination method (Applied Biosystems, Foster City, CA, USA). All genotyping was blinded to clinical variables.

**IFN-λ3 serum levels**

IFN-λ3 serum levels were measured in a randomly selected sub-cohort of 200 subjects to ensure equal distribution of the *IFNL3* genotype and PF, using a highly sensitive chemiluminescent enzyme immunoassay (lifespan bioscience). The sensitivity of the assay was 1 pg/mL with dynamic range; 1-1000 pg/mL.

**Bleomycin-induced pulmonary fibrosis**

C57BL/6J male mice were instilled with 3mg/kg bleomycin into the trachea; control mice were given saline instead of bleomycin. Mice tissues were harvested at 21 days by SMC Laboratories, Inc (Japan).

**RNA extraction and cDNA synthesis**

RNA was extracted using the RNeasy kit (Qiagen) according to the manufacturer’s instructions. RNA quality and concentration was assessed using the Agilent 2100 Bioanalyser (Agilent, Waldbronn, Germany). cDNA was prepared using qscript (Quanta Biosciences, Gaithersburg, MD, USA) in a Mastercycler gradient 5331 (Eppendorf AG, Hamburg, Germany).

**Droplet digital PCR.**

Absolute quantification of Ifnl3 transcripts was performed by ddPCR analysis according to the manufacturer's instructions using a specific probe (Taqman gene expression probe, life technology (Mm00663660_g1), as previously described ([4](#_ENREF_4)). Briefly, reactions were performed in a 20-μl reaction volume that consisted of 10 μl of ddPCR™ Supermix for Probes (186-3010) (Bio-Rad), 1 μl of gene-specific probe, and 9 μl of cDNA sample. Droplet formation was carried out using a QX100 droplet generator (Bio-Rad). Each sample was partitioned into an emulsion of approximately 20,000 uniformly sized nanoliter droplets. The droplet emulsion was transferred to a 96-well plate (Eppendorf) and heat-sealed at 180 °C for 5 s with foil. Thermal cycling was performed using the C1000 Touch Thermal Cycler (Bio-Rad) according to the manufacturer's cycling conditions. Each plate was incubated at 95 °C for 10 min, followed by 40 cycles of 94°C for 30 s and 60 °C for 1 min, with a final 10-min incubation at 98 °C. At least two negative-control wells with no cDNA template were included in every run. After PCR, the RNA content of the droplets was quantified using the QX200 Droplet Reader (Bio-Rad). ddPCR data were analyzed using QuantaSoft software. The fractions of positive and negative droplets were determined, and data were fitted to Poisson statistics and the background was corrected on the basis of the data for the no-template control. Absolute transcript levels are presented as copies per microliter and converted to copies per nanogram of RNA on the basis of the input amount of RNA.


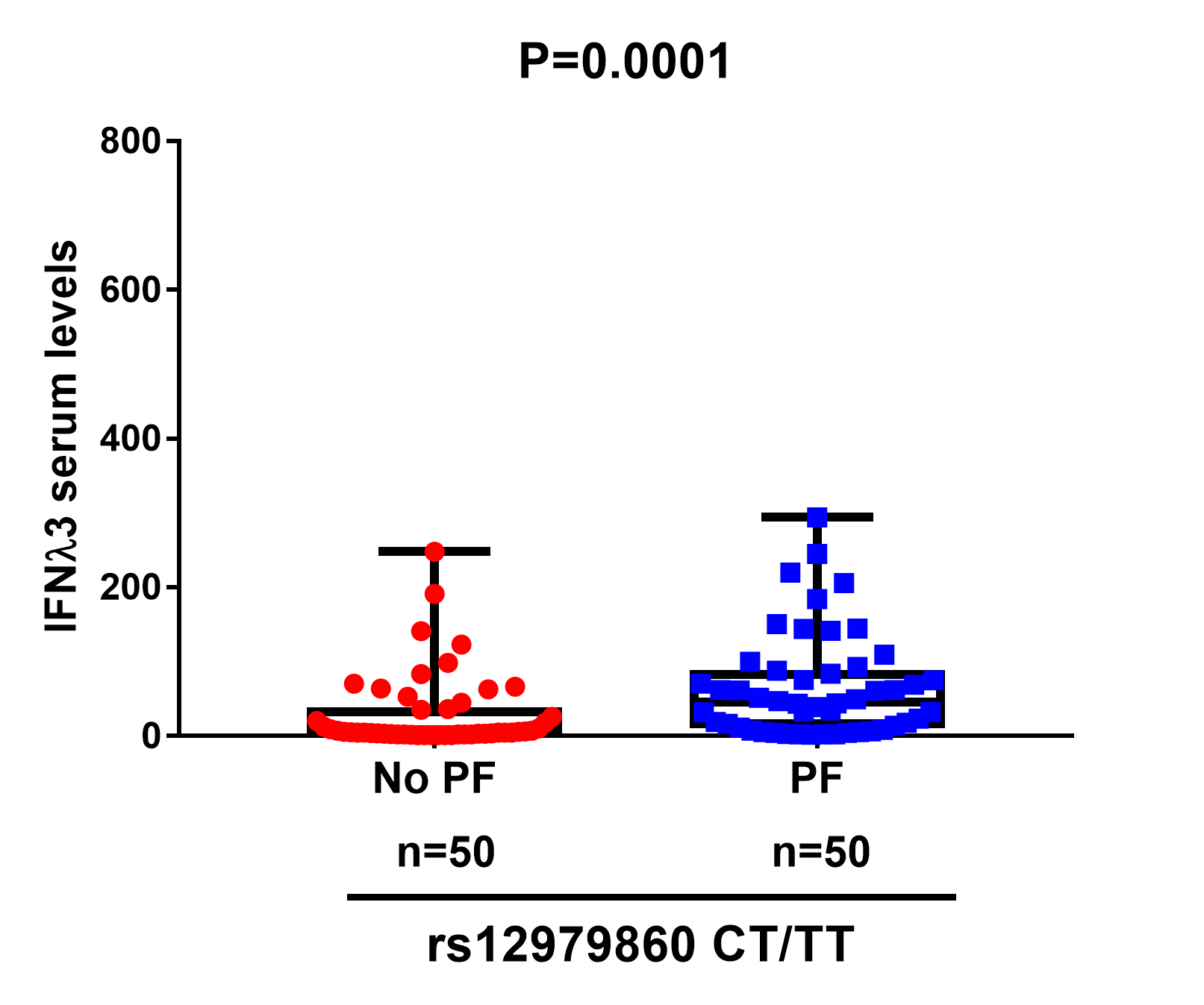

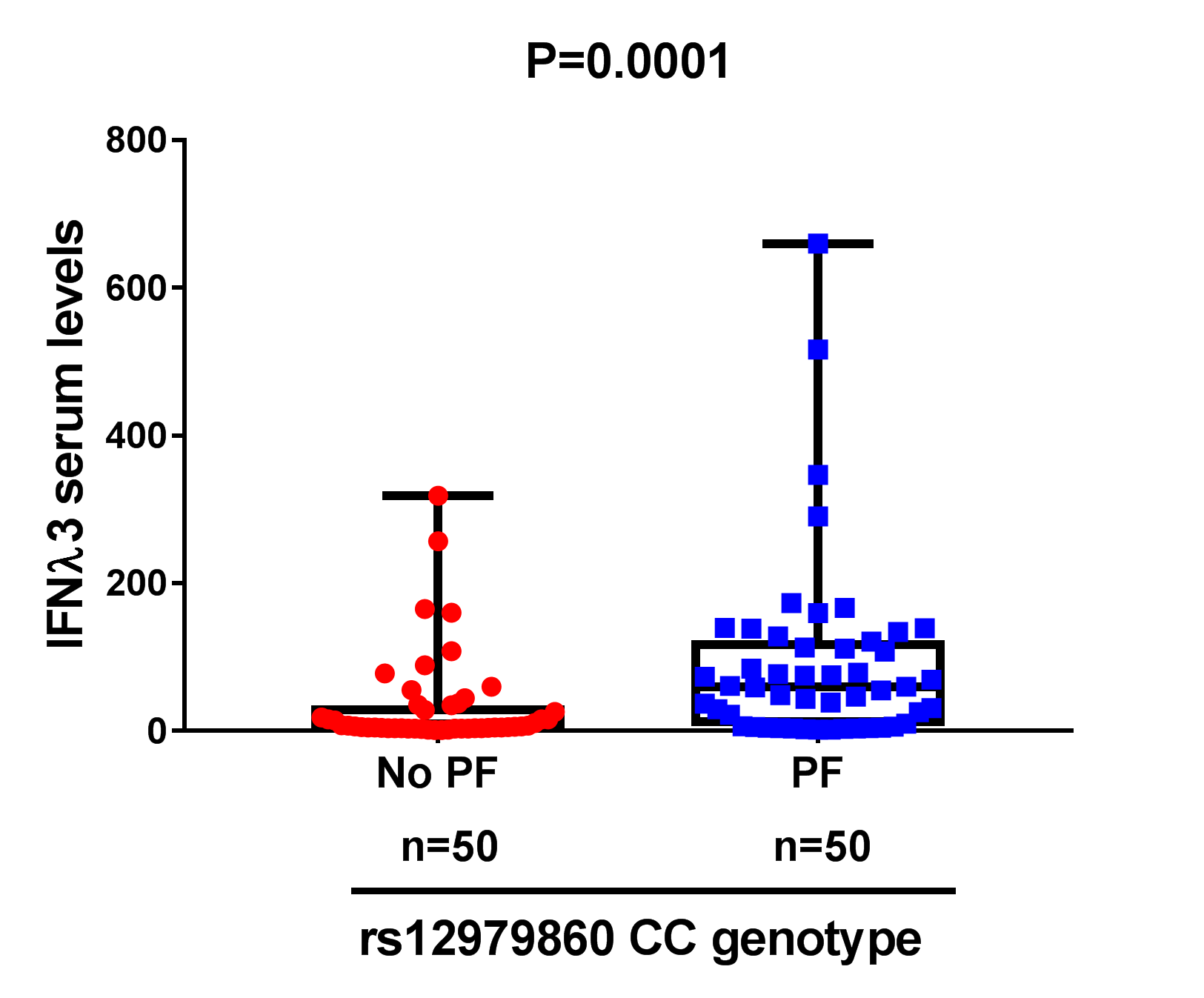


**Supplementary figure 1: quantification of IFNλ3 using a highly sensitive chemiluminescent enzyme immunoassay in human serum samples.** The levels of IFNλ3 were measured in 200 patients with SSc by a chemiluminescent enzyme immunoassay relative to the presence or absence of pulmonary fibrosis (PF). The cohort comprised 100 with rs12979860 genotype CC, and 100 with rs12979860 genotype CT/TT. The x axis shows pulmonary fibrosis status dichotomized as absent (n=100) or present (n=100). The y axis shows serum IFNλ3 levels as pg/mL. The number of independent samples tested in each group is shown in parentheses. Each group is shown as a box plot and the median values are shown as thick dark horizontal lines. The box covers the twenty-fifth to seventy-fifth percentiles. We tested the difference in median values among genotypes using the two-tailed Mann–Whitney test and plotted the box plots using Graph pad prism 7.

**
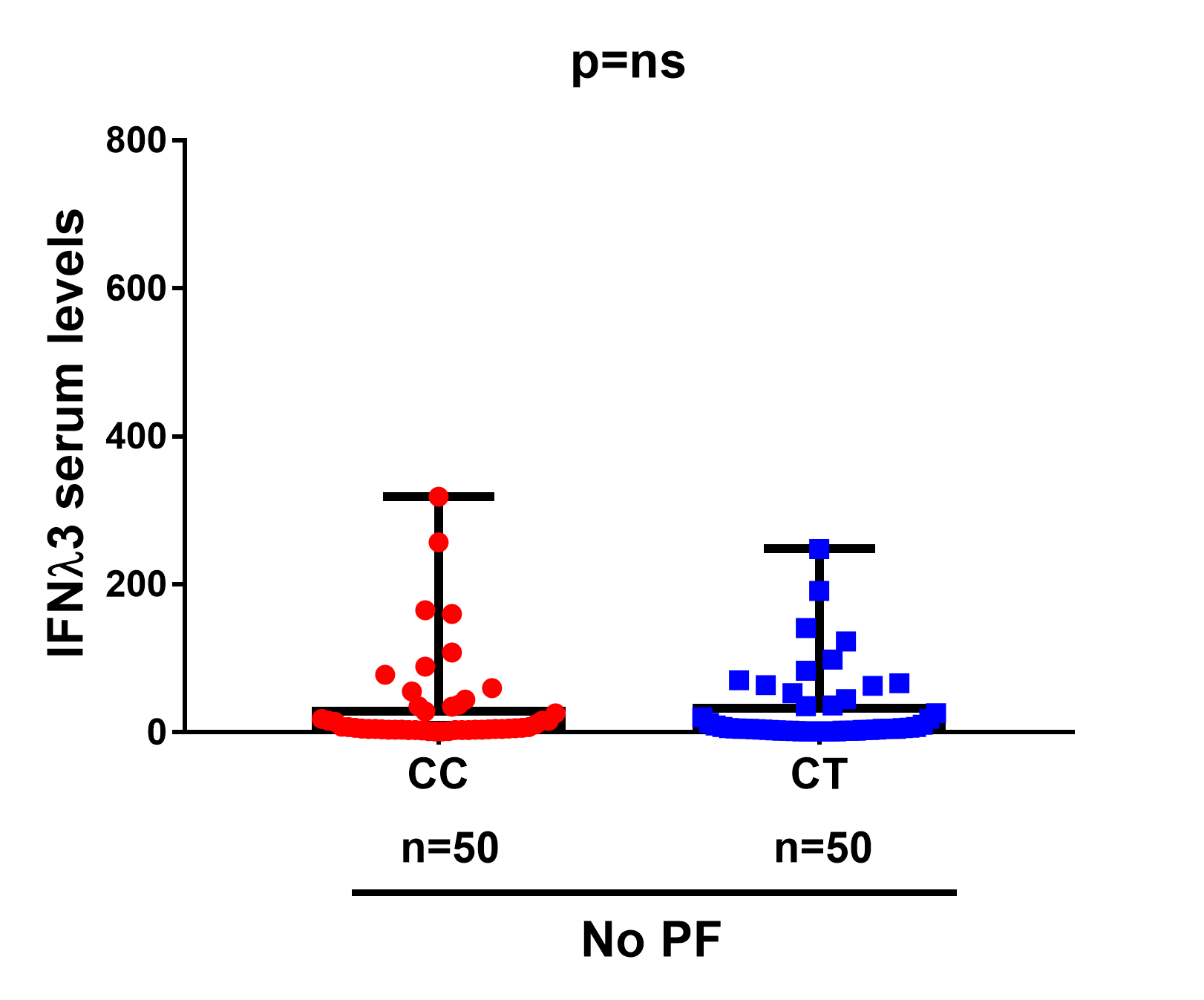

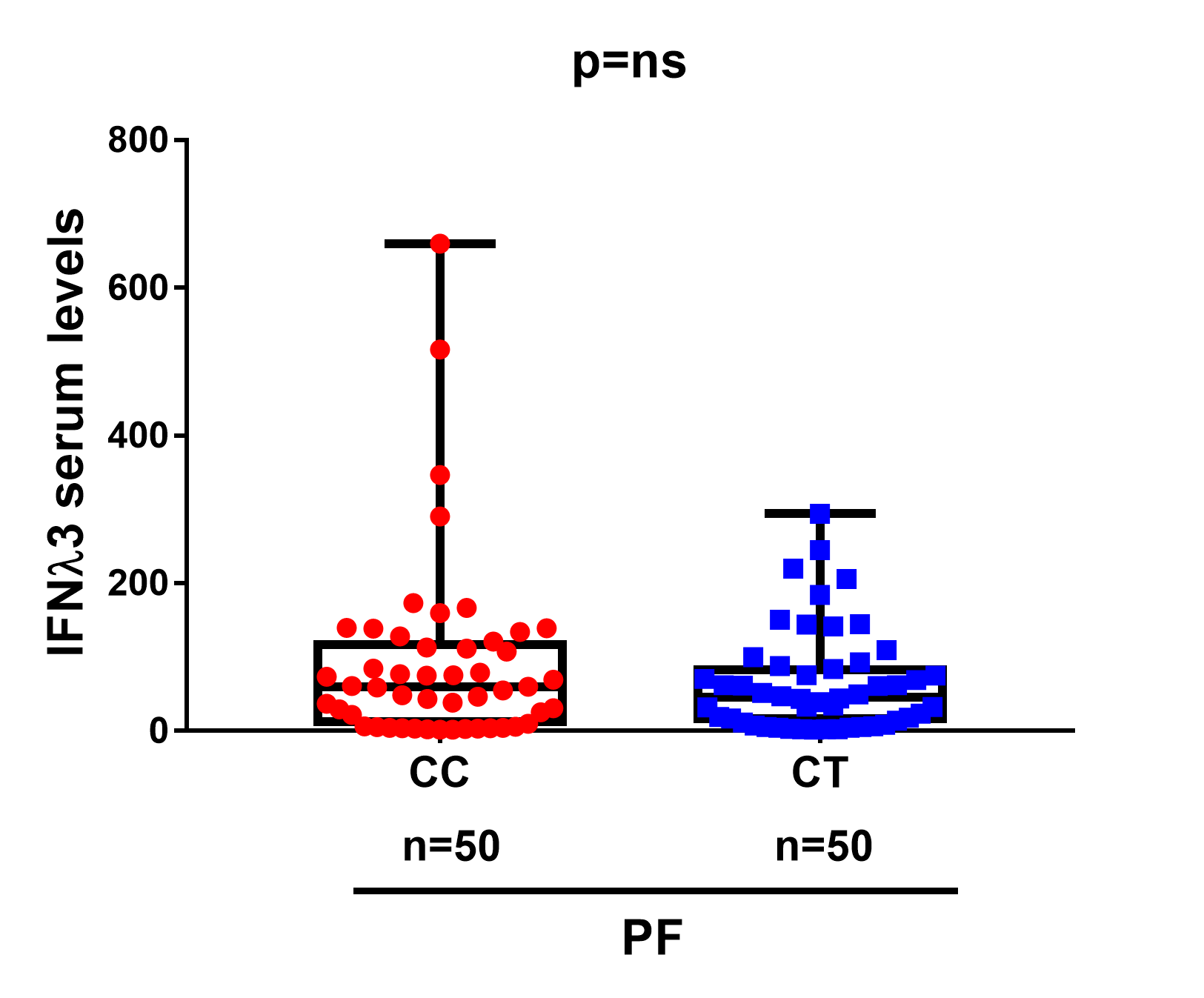
**

**Supplementary figure 2: quantification of IFNλ3 using a highly sensitive chemiluminescent enzyme immunoassay in human serum samples.** The levels of IFNλ3 were measured in 200 patients with SSc by a chemiluminescent enzyme immunoassay relative to rs12979860 CC or CT/TT genotype and stratified according to the presence or absence of pulmonary fibrosis (PF). The x axis shows rs12979860 genotype. The y axis shows serum IFNλ3 levels as pg/mL. The number of independent samples tested in each group is shown in parentheses. Each group is shown as a box plot and the median values are shown as thick dark horizontal lines. The box covers the twenty-fifth to seventy-fifth percentiles. We tested the difference in median values among genotypes using the two-tailed Mann–Whitney test and plotted the box plots using Graph pad prism 7.

| **Variables** | **Patient cohort (n=733)** |
| --- | --- |
| **Age (Years)** | 59 (50.3- 66.4) |
| **Female n (%)** | 631 (86) |
| **Diffuse SSc n (%)** | 155 (21.1) |
| **Lung fibrosis ^1^** | 180 (24.5) |
| **Renal crisis ^2^** | 22 (3) |
| **Laboratory markers** |  |
| ANA | 682 (93) |
| ACA | 361 (49.2) |
| Anti-Scl70 | 93 (12.7) |
| Anti-RNA polymerase III | 61 (8.3) |
| ***IFNL3* rs12979860 genotype^#^** |  |
| CC | 345 (47) |
| CT | 309 (42.1) |
| TT | 76 (10.4) |

**Suplementary table 1: Description of the cohort at baseline**

*^1^Pulmonary fibrosis was defined on high resolution CT chest. ^2^Renal crises were defined as the presence of at least two of the following: new onset hypertension, rising creatinine or microangiopathic haemolytic anemia. P values for Hardy-Weinberg equilibrium were calculated by chi square test and was p=0.7, p >0.05 indicates no deviation from Hardy-Weinberg equilibrium. # Genotype was missing in 3 cases in the cohort.*

**References**

1. Proudman SM, Stevens WM, Sahhar J, Celermajer D. Pulmonary arterial hypertension in systemic sclerosis: the need for early detection and treatment. Intern Med J 2007;37:485-494.

2. Gabrielli A, Avvedimento EV, Krieg T. Scleroderma. N Engl J Med 2009;360:1989-2003.

3. Dobrota R, Maurer B, Graf N, Jordan S, Mihai C, Kowal-Bielecka O, Allanore Y, et al. Prediction of improvement in skin fibrosis in diffuse cutaneous systemic sclerosis: a EUSTAR analysis. Ann Rheum Dis 2016;75:1743-1748.

4. Eslam M, McLeod D, Kelaeng KS, Mangia A, Berg T, Thabet K, Irving WL, et al. IFN-λ3, not IFN-λ4, likely mediates IFNL3–IFNL4 haplotype–dependent hepatic inflammation and fibrosis. Nature Genetics 2017;49:795.
